# Analysis and comparative genomics of R997, the first SXT/R391 integrative and conjugative element (ICE) of the Indian Sub-Continent

**DOI:** 10.1038/s41598-017-08735-y

**Published:** 2017-08-17

**Authors:** Michael P. Ryan, Patricia Armshaw, John A. O’Halloran, J. Tony Pembroke

**Affiliations:** 1 Department of Chemical Sciences, School of Natural Sciences, University of Limerick, Limerick, Ireland; 2 Bernal Institute, University of Limerick, Limerick, Ireland

## Abstract

The aim of this study was to analyse R997, the first integrative and conjugative element (ICE) isolated from the Indian Sub-Continent, and to determine its relationship to the SXT/R391 family of ICEs. WGS of *Escherichia coli* isolate AB1157 (which contains R997) was performed using Illumina sequencing technology. R997 context was assessed by *de novo* assembly, gene prediction and annotation tools, and compared to other SXT/R391 ICEs. R997 has a size of 85 Kb and harbours 85 ORFs. Within one of the variable regions a HMS-1 β-lactamase resistance gene is located. The Hotspot regions of the element contains restriction digestion systems and insertion sequences. R997 is very closely related to the SXT-like elements from widely dispersed geographic areas. The sequencing of R997 increases the knowledge of the earliest isolated SXT/R391 elements and may provide insight on the emergence of these elements on the Indian sub-continent.

## Introduction

Integrative conjugative elements (ICEs) are a class of diverse bacterial mobile elements that are characterized by their ability to mediate and encode all determinants for their own integration, excision, and transfer from one host genome to another by a mechanism of site-specific recombination, self-circularisation, and conjugative transfer^1^. They are a major factor in the evolution of bacterial genomes allowing bacteria to rapidly acquire new phenotypic traits and adaptive functions such as resistance to antimicrobial compounds and heavy metals, virulence mechanisms, metabolic pathways (such as pathways for the degradation of xenobiotic pollutants) and the ability to resist bacteriophage infection^1–4^. SXT/R391 ICEs are chromosomal mobile genetic elements that consist of a conserved integrase that mediates site-specific integration into the 5′ end of the *prfC* gene^5, 6^. The SXT/R391 family of ICEs is one of the largest of the ICE families with >100 elements being identified experimentally or bioinformatically to date^7^. R391 was the first element of the family discovered, in 1967; in a *Providencia rettgeri* clinical isolate from South Africa^8^. The R391 ICE mediates resistance to kanamycin and the heavy metal Hg^9^. In late 1992, SXT in MO10 was first discovered in one of the initial *Vibrio cholerae* O139 clinical isolates from Madras. This *V. cholerae* serogroup was the first non-O1 *V. cholerae* serogroup to give rise to epidemic cholera^10, 11^. SXT^MO10^ is an ~100 kb ICE that carries genes encoding resistance to sulfamethoxazole, trimethoprim, chloramphenicol, and streptomycin^12^. Since then, SXT/R391 like elements have been found in a variety of *Vibrio* species as well as in other Gammaproteobacteria species including Shewanella, Proteus and Photobacterium species (11, Suppl Data).

This family of ICEs contains 51 near identical core genes, many of which are involved in integration/excision, conjugative transfer and regulation of the ICEs^12–15^. In addition, the elements contain five hotspots (called HS1-5) and five variable regions (called VRI-V) where accessory genes, such as antibiotic resistance genes, heavy metal resistant and DNA repair genes, can be found inserted^1, 2, 16^. These elements can also promote the mobilisation of non-transmissible genomic islands and virulence plasmids between hosts^17^.

R997 was the first R391/SXT element to be isolated on the Indian sub-continent. It was identified in 1977 in *Proteus mirabilis* (15 years before the emergence of SXT in MO10) and was found to contain a novel β-lactamase enzyme that was called HMS-1^18^. The element was sequenced in order to gain knowledge of the earliest isolated SXT/R391 elements and their emergence on the Indian sub-continent.

## Results and Discussion

**Figure 1:**
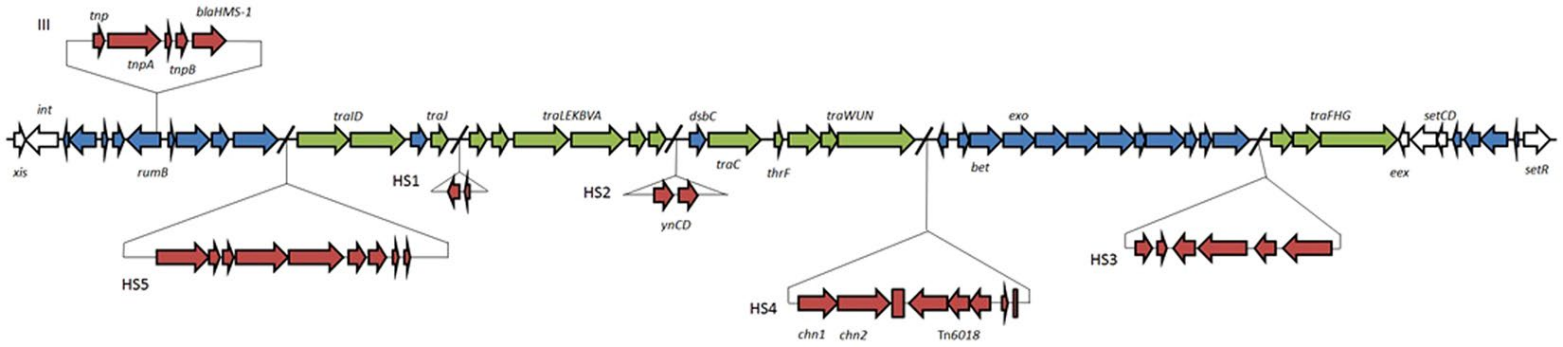
Molecular map of the ICE R997 showing the location of the genes associated with the 85 kb element. Genes in white are involved in R997 integration, excision and control; genes in green are involved in R997 conjugative transfer; genes in blue are involved in other R997 functions, genes in red are the accessory genes.

R997 had 85 Open Reading Frames (ORFs) and followed the conserved synteny for R391/SXT elements (Fig. 1). 51 of these ORFs coded for the core scaffold of R391/SXT elements (genes related to integration, excision and conjugative transfer)^12^. All other genes were found in the hotspot and variable regions of the R997 genome (Fig. 1).

R997 Hotspot 1 (HS1) contains the same two gene insertion as found in HS1 in R391 (orf37 and 38) (showing 97% and 98% nucleotide identity). The functions of these genes are unknown.

R997 Hotspot 2 (HS2) contains the previously described toxin/antitoxin system genes *mosA* and *mosT* that have been experimentally shown to help ICE maintenance within the host organism^19^. The two genes showed 98% and 99% nucleotide identity to the corresponding genes in SXT (s052 and s053).

The insertion in Hotspot 3 (HS3) is made up of six ORFs, the first of which is a truncated version of orf79 of R391. The second ORF is a truncated version of I533_05725 found in ICE*Ama*AS1, while the third ORF is a truncated version of *Val*Spa1_06 found in ICE*Val*Spa1. The next three ORFs are homologs of *Val*Spa1_07, *Val*Spa1_08 and *Val*Spa1_10^20^. These ORFs all code for hypothetical proteins whose functions are unknown.

The insertion in Hotspot 4 (HS4) bore high similarity to an insertion present in HS4 of ICE*Pmi*Chn1^21^. The insertion consisted of three ORFs that code the predicted SIR2 superfamily of proteins: one which catalyses NAD^+^-dependent protein/histone deacetylation, one that codes for a predicted ATPase and one that encodes an Endonuclease I precursor. These ORFs are followed by an insertion sequence that is highly similar to that of Tn*6018* (3,372 bp), which was first characterized in *Pseudomonas putida* (this element was at first described as IS*Ppu*12 but is not an IS and was therefore renamed as Tn6018)^22, 23^. Tn*6018* consists of four ORFs, *cadR-cadA-lspA-tnpA*. *cadA* codes for a putative cation efflux protein^23, 24^, which may provide fitness in the event of exposure to heavy metal. However no heavy metal resistance has previously been reported in conjunction with R997.

The insertion in Hotspot 5 (HS5) showed high similarity to those present in HS5 of ICE*Vch*ChnAHV1003, ICE*Vch*Chn2255 and ICE*Pmi*Chn901-5^25, 26^. The ORFs in this hotspot code for a putative type I restriction-modification system (RM). These systems carry out DNA modification, recombination, and repair and are composed of three polypeptides: R (restriction endonuclease), which recognizes and cut specific DNA sequences; M (modification), which methylates the same sequence to inhibit DNA cleavage and protect the host cell against invasion of foreign DNA; and S (specificity), which determines the specificity of both R and M. These genes may confer protection against bacteriophages, as was demonstrated for other ICEs of the SXT/R391 family from fish-isolated bacteria^20^.

R997 contains no insertions in Variable Regions I, II and IV. The element does however have an insertion in Variable Region III (VRIII). This region contains the HMS-1 class A β-lactamase gene. This type of β-lactamase has similar activity to the TEM-1, TEM-2 and SHV-1 types of β-lactamase^27^. This enzyme mediates resistance to ampicillin, cefoperazone, cefoperazone-sulbactam, cephaloridine, ampicillin-sulbactam, piperacillin, ticarcillin and ticarcillin-clavulanate^18, 27^. This gene has previously been reported in SXT/R391-like elements found in *Proteus* species from China^26^. R997 has previously been found to be from the S entry exclusion group^28^.

**Figure 2:**
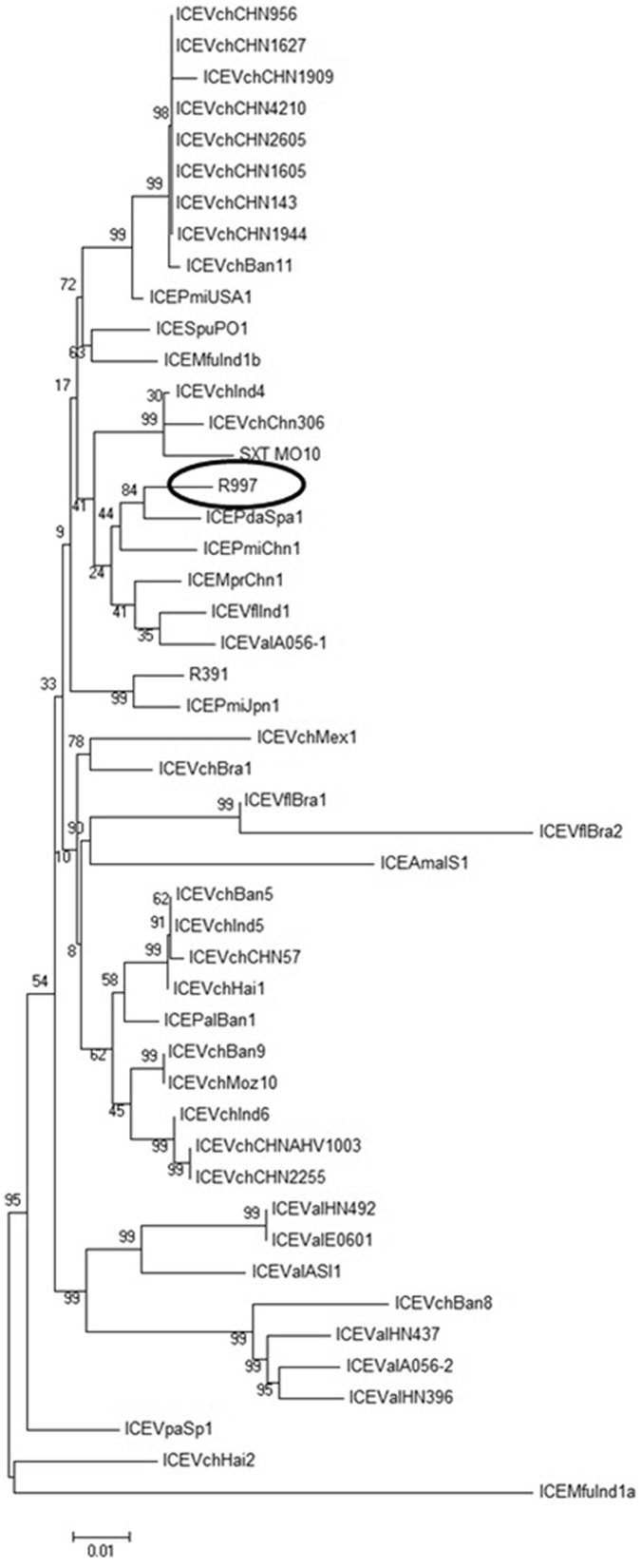
Phylogenetic tree from the maximum-likelihood analysis of the core concatenated proteins of 48 SXT/R391 ICEs.

A phylogenetic tree (Fig. 2) was constructed based on the concatenated amino acid sequences of all SXT-R391 core proteins for all published core genome sequences of these elements. R997 clustered with ICE*Pda*Spa1 which was an ICE contained in *Photobacterium damselae* subsp. *piscicida* PC554.2 isolated from a fish farm in Spain and ICE*Pmi*Chn1, which was an ICE contained in a *P. mirabilis* strain isolated from a chicken in Hubei, China^21^. The element further clustered with ICE*Mpr*Chn1 from *Marinomonas profundimaris* D104 which was isolated from deep-sea sediment in the Arctic Ocean^29^, ICE*Val*A056-1 from *Vibrio alginolyticus* A056 isolated on a shrimp farm in Guangdong, China^30^ and ICE*Vfl*Ind1 from *Vibrio fluvialis* Ind1 isolated in India (12, Supplementary Table 1). These results show the wide geographic spread of SXT/R391 like elements.

R997 was the first SXT/R391 element identified on the Indian Sub-Continent and has not been previously sequenced. This element contains features found in a variety of SXT/R391 elements from around the globe indicating the mosaic nature of these elements. The sequencing of R997 increases the knowledge of the earliest isolated SXT/R391 elements and may provide insight on the emergence of these elements on the Indian sub-continent.

## Methods

### Genome Sequencing and Annotation

The genome of *Escherichia coli* isolate AB1157 (which contains R997) was sequenced by Genospec Inc. (Houston, TX, USA) using paired-end (insert size between the ends 200–500 bp) HiSeq. 2000 Illumina technology giving approximately 300-fold coverage. The resulting reads were processed with Seqprep before being assembled using Newbler v2.5.3. The R997 genome was identified amongst 413 contigs by using the BLAST tool to investigate the presence of several different R391 (AY090559) and SXT (AY055428) core scaffold genes (*int*, *jef*, *traLEKBVA*, *setCD*). The R997 sequence was then annotated using the RAST Server (Rapid Annotation using Subsystem Technology) and the Basic Local Alignment Search Tool (BLAST) programme at NCBI^31, 32^. Any gaps among the sequence were filled in by PCR-linkage and Sanger sequencing. Primers can be seen in Supplementary Table 2. Putative functions for all proteins were inferred using the Basic Local Alignment Search Tool (BLAST) (http://ncbi.nlm.nih.gov/BLAST).

### GenBank Accession Number

R997 was submitted to GenBank under accession number KY433363.

### Phylogenetic Analysis of Core ICE genes

Phylogenetic analysis was performed based on the concatenated amino acid sequences of 48 SXT/R391 core genes encoded proteins on all 47 previously sequenced whole SXT/R391 elements. These elements are listed in Supplementary Table 1. An unrooted phylogenetic tree was constructed by maximum-likelihood method based on the Poisson correction model using the MEGA6^33^. Bootstrap analysis with 1000 replications was performed to test the reliability of the tree.

## Electronic supplementary material

Supplementary Table 1 & 2
