# Supplementary material for: Analysis and comparative genomics of R997, the first SXT/R391 integrative and conjugative element (ICE) of the Indian Sub-Continent
Source: Sci Rep. 2017 Aug 17;7:8562. doi: 10.1038/s41598-017-08735-y (PMC5561048; doi:10.1038/s41598-017-08735-y)
Supplement: Supplementary file 1 — Supplementary Table 1 & 2 [file 41598_2017_8735_MOESM1_ESM.pdf]

Analysis and comparative genomics of R997, the first SXT/R391 integrative and conjugative element (ICE) of the Indian Sub-Continent

Michael P Ryan[1]*, Patricia Armshaw[1, 2], John A. O'Halloran[1, 2] and J Tony Pembroke[1, 2]

[1]Department of Chemical Sciences, School of Natural Sciences, University of Limerick, Limerick, Ireland

[2] Bernal Institute, University of Limerick, Limerick, Ireland

***Corresponding author**:

Michael P Ryan

Department of Chemical Sciences

School of Natural Sciences

University of Limerick

Limerick

Ireland

E-mail: Michael.P.Ryan@ul.ie

Tel No. +353 61 204730

Supplementary Table 1: Identified $ICE_{SXT/R391}$ family members with complete genome sequence available

Element	Strain	Accessory Genes/Reported Functions	Acc. No.	Size (kb)	Ref
ICE R391	*Providencia rettgeri*	Km^r, Hg^r, DNA repair genes, sulphate transporter, toxin-antitoxin system, ATP-dependent Lon protease	AY090559	89	1
ICE SXT (MO10)	*Vibrio cholerae* O139 MO10	Cm^r, Su^r, Tm^r, Sm^r, DNA repair genes, toxin-antitoxin system, ATP-dependent Lon protease, histidine kinase, diguanylate cyclase, deoxycytidine triphosphate deaminase	AY055428	99	2
ICE*Ama*AS1	*Alteromonas macleodii* 'Aegean Sea MED64'	type I RM system	NC_023045	80	3
ICE*Ama*IS1	*Alteromonas macleodii* Ionian Sea UM7	Heavy metal resistance, type I RM system	NC_021713	99	3
ICE*Mfu*Ind1a	*Marinomonas fungiae* JCM 18476	Restriction modification system	LIQF00000000	66	4
ICE*Mfu*Ind1b	*Marinomonas fungiae* JCM 18476	Restriction modification system	LIQF00000000	75	4
ICE*Mpr*Chn1	*Marinomonas profundimaris* D104	Restriction modification system	AYOZ01000000	86	4
ICE*Pal*Ban1	*Providencia alcalifaciens*	Cm^r, Sm^r, Su^r, Tm^r, toxin-antitoxin system, phenazine biosynthesis protein, lysine exporter, glyoxalase resistance, restriction modification system	GQ463139	97	5
ICE*Pda*Spa1	*Photobacterium damselae* subsp. *piscicida* PC554.2	Tc^r, heat-shock protein (dnaK), AAA ATPase, toxin-antitoxin system, ATP-dependent Lon protease ICE*Pmi*USA1	AJ870986	103	6
ICE*Pmi*Chn1	*Proteus mirabilis*	Sm^r, Su^r, Tc^r	KT962845	95	7
ICE*Pmi*Jpn1	*Proteus mirabilis*	Amx^r, Amc^r, Fox^r, Ctx^r	KT894734	93	7
ICE*Pmi*USA1	*Proteus mirabilis strain* HI4320	ATP-dependent helicase, DNA repair proteins, toxin-antitoxin system	AM942759	81	5
ICE*Spu*PO1	*Shewanella putrefaciens* W3-18-1	Cu^r, Zn/Co/Cd RND efflux pump, DNA repair genes, restriction modification system	CP000503	111	8
ICE*Val*A056-1	*Vibrio alginolyticus* A056	Sm^r, Su^r, type III RM system, diguanylate cyclase, acriflavin resistance protein, membrane-fusion protein	KR231688	89	9
ICE*Val*A056-2	*Vibrio alginolyticus* A056	type I RM system, Fic family protein, HigA, MosTA, choline uptake protein, mechanosensitive channel regulation, calcium/sodium proton antiporter, oxaloacetate and citrate metabolism, PrrABCD	KR231689	104	9
ICE*Val*ASI1	*Vibrio alginolyticus* ANC4-19	L-lactate degradation system	NZ_LTYK00000000	N/A	10
ICE*Val*E0601	*Vibrio alginolyticus* E0601	HipBA, Flp pilus assembly system, P pilus assembly system, type III RM system, threonine efflux protein, choline uptake protein, diguanylate cyclase,	KT072768	106	9

Element	Strain	Accessory Genes/Reported Functions	Acc. No.	Size (kb)	Ref
		DDE endonuclease, mechanosensitive channel regulation, RNA-dependent DNA polymerases			
ICE*Val*HN396	*Vibrio alginolyticus* HN396	type II RM system, Fic family protein, HigA, diguanylate cyclase, aerotaxis sensor, chemotaxis sensor, DNA recombination-mediator, DDE endonuclease	KT072770	87	9
ICE*Val*HN437	*Vibrio alginolyticus* HN437	type I RM system, Fic family protein, HigA, organic hydroperoxide resistance, DDE endonuclease, nucleotide metabolism, phage lysin, rhamnose metabolism, sulfate assimilation, synthesis of capsular polysaccharide	KT072771	94	9
ICE*Val*HN492	*Vibrio alginolyticus* HN492	HipBA, Flp pilus assembly system, P pilus assembly system, type III RM system, threonine efflux protein, choline uptake protein, diguanylate cyclase, DDE endonuclease, mechanosensitive channel regulation, RNA-dependent DNA polymerases	KT072769	106	9
ICE*Vch*Ban11	*Vibrio cholerae* 4672	Type II restriction system	ERS016137	96	11
ICE*Vch*Ban5	*Vibrio cholerae* O1	Cm^r, Sm^r, Su^r, Tm^r, glyoxalase resistance, toxin-antitoxin system	GQ463140	102	5
ICE*Vch*Ban8	*Vibrio cholerae* O37 strain MZ03	Toxin-antitoxin system, toxin coregulated pilus, acriflavine resistance	JQ345361	103	5
ICE*Vch*Ban9	*Vibrio cholerae* O1	Sm^r, Su^r, Tm^r, Tc^r,	CP001485	106	5
ICE*Vch*Bra1	*Vibrio cholerae* VCC19	restriction modification system	NZ_ATEV00000000	N/A	12
ICE*Vch*Chn0143	*Vibrio cholerae* ICDC-VC0143	Sm^r, Su^r, Tm^r, Tc^r, toxin-antitoxin system	KT151654	87	13
ICE*Vch*Chn0956	*Vibrio cholerae* ICDC-VC956	Sm^r, Su^r, Tm^r, toxin-antitoxin system	KT151655	94	13
ICE*Vch*Chn1605	*Vibrio cholerae* ICDC-VC1605	Sm^r, Su^r, Tm^r, toxin-antitoxin system	KT151656	98	13
ICE*Vch*Chn1627	*Vibrio cholerae* ICDC-VC1627	Sm^r, Su^r, Tm^r, Tc^r, toxin-antitoxin system	KT151657	92	13
ICE*Vch*Chn1909	*Vibrio cholerae* ICDC-VC1909	Sm^r, Su^r, Tm^r, toxin-antitoxin system	KT151658	93	13
ICE*Vch*Chn1944	*Vibrio cholerae* ICDC-VC1944	Sm^r, Su^r, Tm^r, toxin-antitoxin system	KT151659	89	13
ICE*Vch*Chn2255	*Vibrio cholerae* ICDC-VC2255	Sm^r, Su^r, Tm^r, Tc^r ,type I RM system, toxin-antitoxin system	KT151660	95	13
ICE*Vch*Chn2605	*Vibrio cholerae* ICDC-VC2605	Sm^r, Su^r, Tm^r, toxin-antitoxin system	KT151661	87	13
ICE*Vch*Chn*306*	*Vibrio cholerae* E306	toxin-antitoxin system, ATP-dependent Lon protease	NZ_AWWA01000000	N/A	14
ICE*Vch*Chn4210	*Vibrio cholerae* ICDC-	Sm^r, Su^r, Tm^r, Tc^r, toxin-antitoxin system	KT151662	110	13

Element	Strain	Accessory Genes/Reported Functions	Acc. No.	Size (kb)	Ref
	VC4210				
ICE*Vch*Chn57	*Vibrio cholerae* ICDC-VC57	Sm^r, Su^r, Tm^r, Tc^r, toxin-antitoxin system	KT151664	93	13
ICE*Vch*ChnAHV1003	*Vibrio cholerae* AHV1003	Ery^r, Sm^r, Su^r, Tm^r, type I RM system, toxin-antitoxin system,	KT151663	102	13
ICE*Vch*Hai1	*Vibrio cholerae* VC1786	Tm^r, Su^r, Sm^r, glyoxalase resistance, toxin-antitoxin system	JN648379	98	15
ICE*Vch*Hai2	*Vibrio cholerae* HC-1A2	N/A	AJRO01000008	84	16
ICE*Vch*Ind4	*Vibrio cholerae* 0139	Cm^r, Sm^r, Su^r, Toxin-antitoxin system, histidine kinase, diguanylate cyclase, ATP-dependent Lon protease	GQ463141	95	5
ICE*Vch*Ind5	*Vibrio cholerae* O1	Cm^r, Sm^r, Su^r, Tm^r, glyoxalase resistance, toxin-antitoxin system	GQ463142	98	5
ICE*Vch*Ind6	*Vibrio cholerae* 4605	Tm^r	ERS013257	90	11
ICE*Vch*Mex1	*Vibrio cholerae* non O1-0139	Fic family protein, diguanylate cyclase, restriction modification system, toxin-antitoxin system, histidine kinase, aminoglycoside resistance, acetyltransferase	GQ463143	83	17
ICE*Vch*Moz10	*Vibrio cholerae* B33	Sm^r, Su^r, Tc^r, toxin-antitoxin system	ACHZ00000000	104	5
ICE*Vfl*Bra1	*Vibrio fluvialis* 560	restriction modification system	JQHX00000000	N/A	18
ICE*Vfl*Bra2	*Vibrio fluvialis* 539	restriction modification system	JQHW00000000	N/A	18
ICE*Vfl*Ind1	*Vibrio fluvialis*	Cm^r, Sm^r, Su^r, Tm^r, toxin-antitoxin system, histidine kinase, diguanylate cyclase, restriction modification system, deoxycytidine triphosphate deaminase protein	GQ463144	114	5

Km^r: Kanamycin resistance, Hg^r: Mercury resistance, Su^r: Sulfamethoxazole resistance, Tm^r: Trimethoprim resistance, Sm^r: Streptomycin resistance, Cm^r: Chloramphenicol resistance, Cu^R: Copper resistance, RND: Resistance Nodulation Cell Division, Tc^r: Tetracycline resistance, Zn: Zinc, Co: Cobalt, Cd: Cadmium, AAA: ATPases Associated with diverse cellular activities. N/A: Not Available

In some of these cases no name was given to the ICE that was sequenced. In these cases names were assigned based on the nomenclature system used in Burrus *et al*. (19).

1. Boltner D, MacMahon C, Pembroke JT *et al.* R391: a conjugative integrating mosaic comprised of phage, plasmid, and transposon elements. *J Bacteriol* 2002; **184**: 5158-69.
2. Beaber JW, Hochhut B, Waldor MK. Genomic and Functional Analyses of SXT, an Integrating Antibiotic Resistance Gene Transfer Element Derived from *Vibrio cholerae*. *J Bacteriol* 2002; **184**: 4259-69.
3. López-Pérez M, Gonzaga A, Rodriguez-Valera F. Genomic Diversity of "Deep Ecotype" *Alteromonas macleodii* Isolates: Evidence for Pan-Mediterranean Clonal Frames. *Genome Biol Evol* 2013; **5**: 1220-32.
4. Badhai J, Das SK. Characterization of Three Novel SXT/R391 Integrating Conjugative Elements ICE*Mfu*Ind1a and ICE*Mfu*Ind1b, and ICE*Mpr*Chn1 Identified in the Genomes of *Marinomonas fungiae* JCM 18476(T) and *Marinomonas profundimaris* Strain D104. *Front Microbiol* 2016; **7**: 1896.
5. Wozniak RA, Fouts DE, Spagnoletti M *et al.* Comparative ICE genomics: insights into the evolution of the SXT/R391 family of ICEs. *PLoS Genet* 2009; **5**: e1000786
6. Osorio CR, Marrero J, Wozniak RAF *et al.* Genomic and Functional Analysis of ICE*Pda*Spa1, a Fish-Pathogen-Derived SXT-Related Integrating Conjugative Element That Can Mobilize a Virulence Plasmid. *J Bacteriol* 2008; 190: 3353-61.
7. Lei C-W, Zhang A-Y, Wang H-N *et al.* Characterization of SXT/R391 Integrative and Conjugative Elements in *Proteus mirabilis* Isolates from Food-Producing Animals in China. *Antimicrob Agents and Chemother* 2016; **60**: 1935-8.
8. Pembroke JT, Piterina AV. A novel ICE in the genome of *Shewanella putrefaciens* W3-18-1: comparison with the SXT/R391 ICE-like elements. *FEMS Microbiol Lett* 2006; **264**: 80-8.
9. Luo P, He X, Wang Y *et al.* Comparative genomic analysis of six new-found integrative conjugative elements (ICEs) in *Vibrio alginolyticus. BMC Microbiol* 2016; **16**: 79.
10. Bhotra T, Singh DV. Whole-Genome Sequence of *Vibrio alginolyticus* Isolated from the Mucus of the Coral *Fungia danai* in the Andaman Sea, India. *Genome Announc* 2016; **4**: e00339-16.
11. Spagnoletti M, Ceccarelli D, Rieux A *et al.* Acquisition and Evolution of SXT-R391 Integrative Conjugative Elements in the Seventh-Pandemic *Vibrio cholerae* Lineage. *mBio* 2014; **5**.
12. de Sá PCG, Da Silva ML, Carneiro AR *et al.* Draft Genome Sequence of Non-O1 and Non-O139 *Vibrio cholerae* Strain VCC19. *Genome Announc* 2014; **2**: e01094-14.
13. Wang R, Yu D, Yue J et al. Variations in SXT elements in epidemic *Vibrio cholerae* O1 El Tor strains in China. *Sci Rep* 2016; **6**: 22733.
14. Yi Y, Lu N, Liu F *et al.* Genome sequence and comparative analysis of a *Vibrio cholerae* O139 strain E306 isolated from a cholera case in China. *Gut Pathog* 2014; **6**: 3-.
15. Sjölund-Karlsson M, Reimer A, Folster JP *et al.* Drug-Resistance Mechanisms in *Vibrio cholerae* O1 Outbreak Strain, Haiti, 2010. *Emerg Infect Diseases* 2011; **17**: 2151-4.
16. Ceccarelli D, Spagnoletti M, Hasan NA *et al.* A new integrative conjugative element detected in Haitian isolates of *Vibrio cholerae* non-O1/non-O139. *Res Microbiol* 2013; **164**: 891-3.
17. Burrus V, Quezada-Calvillo R, Marrero J *et al.* SXT-Related Integrating Conjugative Element in New World *Vibrio cholerae. Appl Environ Microbiol* 2006; **72**: 3054-7.

18. de Oliveira Veras AA, da Silva ML, Gomes JCM *et al*. Draft Genome Sequences of *Vibrio fluvialis* Strains 560 and 539, Isolated from Environmental Samples. *Genome Announc* 2015; **3**: e01344-14.
19. Burrus V, Marrero J, Waldor MK. The current ICE age: Biology and evolution of SXT-related integrating conjugative elements. *Plasmid* 2006; 55: 173-83.

Supplementary Table 2: Primers to close gaps in R997 Sequence

Name		Gap Closed	Sequence
316	FW	VRIII Region	GAGTACGCAAGTCATTGGCG
317	REV		CACCCAGACTGAAGCTTCG
330	FW	HS4 Region	CGCCCTCTCGATGTTTGGA
331	REV		GATGGACTGTCCATCAGAAGAAC
342	FW	HS5 Region	GGTTACGGTTTTGCA
343	REV		CTTAAGGCACTGTCA
